# An Appraisal of Female Sex Work in Nigeria - Implications for Designing and Scaling Up HIV Prevention Programmes

**DOI:** 10.1371/journal.pone.0103619

**Published:** 2014-08-13

**Authors:** Akudo Ikpeazu, Amaka Momah-Haruna, Baba Madu Mari, Laura H. Thompson, Kayode Ogungbemi, Uduak Daniel, Hafsatu Aboki, Shajy Isac, Marelize Gorgens, Elizabeth Mziray, Ndella Njie, Francisca Ayodeji Akala, Faran Emmanuel, Willis Omondi Odek, James F. Blanchard

**Affiliations:** 1 National Agency for the Control of AIDS, Abuja, Nigeria; 2 Centre for Global Public Health, Department of Community Health Sciences, University of Manitoba, Winnipeg, Canada; 3 World Bank, Washington, DC, United States of America; University of Washington, United States of America

## Abstract

**Background:**

The HIV epidemic in Nigeria is complex with diverse factors driving the epidemic. Accordingly, Nigeria's National Agency for the Control of AIDS is coordinating a large-scale initiative to conduct HIV epidemic appraisals across all states. These appraisals will help to better characterize the drivers of the epidemic and ensure that the HIV prevention programmes match the local epidemic context, with resources allocated to interventions that have the greatest impact locally. Currently, the mapping and size estimation of Female Sex Workers (FSWs) - a major component of the appraisal has been completed in seven states. These states are using the data generated to plan, prioritize and scale-up sub-national HIV prevention programmes.

**Methodology:**

It involved a two-level process of identifying and validating locations where FSWs solicit and/or meet clients (“hotspots”). In the first level, secondary key informants were interviewed to collect information about the geographic location and description of the hotspots. For the second level, FSWs were interviewed at each hotspot and information on population size estimates, typologies and operational dynamics of the FSWs were collected.

**Results:**

Across the seven states, a total of 17,266 secondary key informants and 5,732 FSWs were interviewed. 10,233 hotspots were identified with an estimated 126,489 FSWs ranging from 5,920 in Anambra to 46,691 in Lagos. The most common hotspots were bars/nightclubs (30%), hotels/lodges (29.6%), streets (16.6%), and brothels (14.6%). Furthermore, the population density of FSWs (per thousand adult men) across the states ranged from 2 in Anambra to 17 in the Federal Capital Territory.

**Conclusion:**

FSW populations in Nigeria are large and diverse, with substantial differences between and within states. Improved understanding of the location, population size, density, organizational typologies and clients of sex work has informed and is central to Nigeria's planning process for scaling up focused HIV prevention programmes.

## Introduction

Policy makers and programme implementers require accurate and timely guidance on the status and key epidemiologic drivers of ongoing local transmission to ensure that they maximize the impact of their investments in HIV prevention [1]. This is especially relevant in a country such as Nigeria with the second largest number of new HIV infections globally (nearly 300,000 annually) and substantial heterogeneity in HIV prevalence across different regions with diverse factors that drive the epidemic locally [2].

Given that an effective HIV prevention strategy must be guided by information about the underlying epidemiologic drivers and proximal sources of new infections, it is imperative that epidemic appraisals are conducted within local contexts to facilitate the identification of these relevant drivers of local HIV transmission [1]. In addition, there is growing evidence on the use of geographic clustering of HIV infections as a means to identify populations at higher risk of HIV infection, and subsequently targeting HIV prevention efforts on priority geographic areas to maximize the impact on the HIV epidemic [3]. In Nigeria, it is estimated that the key populations considered to be most at risk for HIV transmission, Female Sex Workers (FSWs), Injecting Drug Users, and Men who have sex with Men and their partners contribute as much as 40% of new HIV infections, despite representing only about 3.4% of the adult population. Half of the infections contributed by these key populations and their partners are attributed to FSWs, their clients and clients' partners alone, highlighting a profound need for programmatic response focus on this sub-population group [4], [5]. This is not surprising given the low levels of risk perception (39%) and knowledge about HIV (31%) reported among FSWs [6]. Furthermore, it has been well documented that HIV transmission in the context of sex work plays a significant role in the expansion of HIV epidemics in many countries around the world [7], [8], [9].

In Nigeria, the national Integrated Biological and Behavioral Surveillance Survey (IBBSS) conducted across nine states reached FSWs by visiting hotspots identified by the State Agencies for Control of AIDS (SACAs) and Non-Governmental Organizations as locations where FSWs congregate. While this national survey did not derive the population size estimates of the FSW at these locations, it showed that the HIV prevalence among brothel-based FSWs in Nigeria varied considerably. It ranged from a low of 12% in Lagos state to a high of about 46% in Benue and Nasarawa state, incidentally states that also have high ANC prevalence (12.7% and 7.5% respectively) [5]. This heterogeneity may be partially explained by regional differences in socioeconomic and cultural factors which may influence empowerment, opportunities, and stigma of FSWs, which in turn influence vulnerability to HIV infection [10]. Various prevention programmes have been implemented for FSWs by both governmental and nongovernmental organizations in Nigeria. These include programmes that are designed to increase their knowledge of HIV, increase condom use with clients through provision of free condoms and condom negotiation skills and offer free HIV voluntary counseling and testing (VCT) [6], [11], [12]. Some of the key challenges for program design have been the lack of available information about the size and specific locations of key target populations, lack of a standardized, integrated package of services and poor coordination among implementers – all of which would be useful for determining the necessary scale and reach of HIV prevention programmes in order to maximize their impact.

Nigeria's National Agency for the Control of AIDS (NACA) is coordinating a large-scale national initiative to conduct rapid HIV epidemic appraisals across all states, which includes the mapping and size estimation of FSWs. The other components of the appraisal, Venue Profiling and Rural Appraisals were also conducted with the view of assessing the sexual behavior of other vulnerable populations in urban settings and the general population in rural settings respectively given that these target groups are also significant contributors to Nigeria's mixed epidemic. In this paper, we describe how the information obtained from the appraisal is providing the SACAs in Anambra, Benue, Cross River, Federal Capital Territory (FCT), Lagos, Nasarawa, and Ondo states with critical intelligence to plan, prioritise and scale up HIV prevention interventions for FSWs. These seven states pioneered the local epidemic appraisals in Nigeria.

## Methods

A “female sex worker” was defined as any female who receives money or other valuable gifts/incentives from a man in exchange for sex in areas such as brothels, bars, hotels, nightclubs, restaurants, or on the street [6]. The rationale behind the mapping methodology that the Government adopted was based largely on the understanding that most FSWs, particularly those who are most active, congregate and/or meet clients in definable geographic locations. Accordingly, the Government's approach entailed focusing on identifying these locations, characterizing each location in terms of specific “spots” within that location and the operational characteristics of sex work there (i.e. how and where FSWs meet clients and where transactions occur), and estimating the number of FSWs that frequent the location and spots. This approach is an adaptation of the PLACE (priorities for local AIDS control efforts) methodology, which identified locations where individuals frequented to acquire new sexual partners [13], [14].

To ensure the methodology was applicable to the local context, the National Prevention Technical Working Group held a series of consultative meetings to reach consensus on the methodology and instruments to be used for the geographic mapping. Thereafter, a core team of master trainers from each state was trained on the geographic mapping approach and they in turn trained their field teams at state level with support from the technical team. The field operations of the geographic mapping comprised of two major phases, referred to as “levels”. In Level 1, secondary key informants – people knowledgeable about the area (e.g., taxi drivers, commercial motorcyclists, shopkeepers etc) were interviewed about the geographic locations (“hot spots”) where FSWs congregate and solicit clients. To facilitate Level 1 data collection, all of the states except FCT and Lagos selected major towns in the state and divided these towns into smaller zones based on population estimates, physical features and landmarks. Lagos and FCT were divided into zones, as there are no well-defined towns in these states. Typically, 60 key informant interviews were conducted in each zone with a total of 17,266 interviews conducted across the seven states. The product of Level 1 activity was a comprehensive list of unique spots where FSWs may be found, the typology of the spot (e.g. brothels, street, hotels etc) and estimated minimum, and maximum numbers of FSWs at each spot.

This information was validated in Level 2 by interviewing 5732 FSWs at the identified hotspots. A few FSWs were recruited in each of the towns mapped to assist data collectors with identifying the members of their groups at the identified spots for validation interviews. The validation process determined the existence of a spot, whether or not the spot was frequented by FSWs, the estimated minimum and maximum number of FSWs who frequented active spots on usual and peak days and the operational dynamics of each spot (peak and non-peak times). In the validation, spots that were mentioned by the least number of secondary key informants at Level 1 were given priority, because these were the most likely to have been incorrectly identified.

To generate the population size estimate of FSWs at each spot, the average of the minimum and maximum number of FSWs that frequented each spot was derived. For the spots validated in Level 2, only validated data were used in generating population size estimates while for those spots not re-visited, an average of the minimum and maximum population size estimates obtained from secondary key informants in Level 1 were used. These average population size estimates obtained from Level 1 were adjusted by applying the correction factor derived by analyzing the differences in the estimate given by secondary key informants (level 1) and spot validation data (level 2) for the validated spots.

Level 2 interviews also sought other locations in the vicinity not identified by Level 1 secondary key informants. Following the validation of the hotspot, the interviewer obtained the GIS coordinates of the location, which were used for the spatial representation of the hotspot on the map of the state. The FSW density per 1000 adult males was obtained by dividing the FSW population estimate by the total population of adult males (>18 years) in the state.

### Ethics statement

Interviewers were required to read out a consent form to each participant. This form explained the objectives of the study, enquired as to whether the participant was willing to respond to the questions and required him/her to indicate their agreement through verbal consent. Participants provided verbal informed consent to participate in the study. They were not required to disclose any personal information and no biological specimen was obtained. The study protocol and consent procedure was reviewed and approved by the State Ministry of Health's Ethical Committee in Anambra, Cross River, Benue, Nasarawa, FCT, Lagos and Ondo states.

### Data analysis

Data were collected from April to June 2012 across the seven states and processing was done using a Microsoft Access database with in-built quality checks. The same software was used to generate a list of unique spots and calculate FSW population size estimates by spot typology, zone and town. The analysis provided minimum and maximum estimates for each spot and to arrive at a point estimate, averages of the minimum and maximum estimates were calculated.

## Results

### Key informant interviews

A total of 17,266 secondary key informants and 5732 primary key informants (FSWs) were interviewed to identify spots frequented by FSWs, with more than 1,000 key informants per state (Table 1). The secondary key informants were predominantly male (75%) and 78 per cent of them had secondary or higher level of education. They included petty shop owners (12%), and taxi/commercial motorcycle drivers (21%) while majority were involved in “other” professions (46%) (Table 2).

**Table 1:** Number of Key Informant Interviews by State, Sex and Educational level.

State	Number of KI Interviews	Sex		Education Level				
		Male	Female	Primary	Secondary/vocational	Tertiary	None	Did not disclose
FCT	2190	73%	27%	10%	53%	20%	3%	14%
ANAMBRA	1672	64%	36%	27%	51%	15%	3%	4%
BENUE	1844	76%	24%	15%	55%	22%	5%	3%
CROSS RIVER	2308	74%	26%	13%	61%	24%	1%	1%
LAGOS	4940	82%	18%	14%	75%	8%	2%	1%
NASARAWA	2811	79%	21%	13%	52%	21%	13%	0%
ONDO	1501	68%	32%	12%	58%	26%	3%	1%
Total	17266	76%	24%	14%	61%	17%	4%	3%

**Table 2:** Number of key informants by type of key informant and by state.

	Type of Key Informant						
State	Taxi drivers/Commercial motorcyclists	Local food vendors	Security staff	Bar workers	Service providers	Gov/law enforcement officials	Others
FCT	17%	5%	4%	5%	15%	4%	53%
Anambra	16%	8%	2%	6%	16%	2%	49%
Benue	29%	8%	2%	3%	7%	5%	44%
CrossRiver	28%	7%	2%	6%	14%	5%	37%
Lagos	23%	7%	4%	6%	8%	1%	48%
Nasarawa	18%	6%	1%	1%	13%	7%	52%
Ondo	13%	5%	1%	4%	13%	7%	34%
Total	21%	6%	3%	5%	12%	4%	46%

### Female sex worker spots

In Level 2, 23.5% of the hotspots identified by secondary key informants in Level 1 were found to be either non-existent or not frequented by FSWs. A total of 10,233 active FSW spots were identified through the mapping exercise in Level 2, with the fewest in Anambra (618, 6%) and the most in Lagos (4,056, 39.6%) (Table 3).

**Table 3:** Number of FSW spots by state.

State	Number of spots
Anambra	618
Cross River	692
Benue	825
FCT	1146
Ondo	1187
Nasarawa	1409
Lagos	4065
**Total number of active spots**	**10233**

### FSW population estimates

There were an estimated 126,489 FSWs in the 7 states, ranging from 5,920 in Anambra to 46,691 in Lagos (Table 4). Out of the 46,491 FSWs in Lagos, the majority (16%) were found in Alimosho, followed by Agege (8.2%) and Surulere (6.7%) Local Government Areas (LGAs). In FCT, a significant majority (70%) of FSWs were found in AMAC, which encompasses the Central Business District, followed by Gwagwalada (16%). In Nasarawa, the LGAs with the highest number of FSWs were Karu (5404, 27%) and Lafia (3,695, 19%). Markurdi with an estimated 1,962 FSWs accounted for 20 per cent of the FSWs in Benue, followed by Vandekiya and Gboko each accounting for 12 percent of the estimated FSW population. In Cross River, the majority of FSWs were in Calabar Municipality (2,472, 25%) and Ikom (1,112, 11%). Akure South LGA accounted for 28 per cent (n = 2,687) while Odigbo (n = 2,383) and Ondo West (n = 2006) LGAs accounted for 24 and 21 per cent, respectively, of the FSWs in Ondo. In Anambra, Onitsha north LGA had the largest FSW population estimate (1406, 24%) followed by Akwa South (1269, 21%) LGAs. Table 4 also shows that the estimated number of FSWs per 1000 adult males varied across the states with Anambra having the smallest estimated number of FSWs per 1000 adult males (8) and FCT had the highest ratio with an estimated 69 FSWs per 1,000 adult males. There was also considerable heterogeneity in total FSW estimates and estimated number of FSWs per 1,000 adult males between LGAs within a state. In the Federal Capital Territory (FCT), Abaji had the lowest FSW estimate (245) with an estimated 17 FSWs per 1,000 adult males while Abuja Municipal Council (AMAC) had the highest FSW estimate (17,117) with an estimated 88 FSWs per 1,000 adult males. Overall, Njikoka Local Government Area (LGA) in Anambra state had the lowest FSW estimate (61) with an estimated 2 FSWs per 1,000 adult males while Abuja Municipal Council (AMAC) in the FCT had the highest FSW estimate (17,117) with an estimated 88 FSWs per 1,000 adult males.

**Table 4:** Estimated number of FSWs by state, by LGA and average number of FSWs/1000 adult men per state, Nigeria, 2012.

State	LGA	Estimated number of FSWs	Average number of FSWs/1000 adult men (2006)
FCT	ABAJI	245	17
	BWARI	1317	23
	KUJE	1125	46
	AMAC	17117	88
	GWAGWALADA	3928	99
	KWALI	644	30
**FCT**	**Total**	**24376**	**69.3**
ANAMBRA	AGUATA	97	1
	EKWUSIGO	183	5
	NJIKOKA	61	2
	OGBARU	95	2
	ONITSHA SOUTH	188	5
	ORUMBA SOUTH	117	3
	AWKA NORTH	587	21
	IDEMILI NORTH	379	4
	IHIALA	443	6
	NNEWI NORTH	766	20
	ORUMBA NORTH	331	8
	AWKA SOUTH	1269	27
	ONITSHA NORTH	1406	45
**ANAMBRA**	**Total**	**5920**	**8.7**
BENUE	KWANDE	554	9
	OKPOKWU	430	10
	GBOKO	1229	14
	GWER-EAST	873	21
	KATSINA-ALA	760	13
	TAKAR	377	19
	UKUM	895	16
	MAKURDI	1962	26
	OTUKPO	1715	26
	VANDEKYA	1241	21
**BENUE**	**Total**	**10034**	**17.6**
CROSS RIVER	ABI	327	9
	AKPABUYO	315	5
	BIASE	238	6
	BOKI	423	9
	OBANLIKWU	134	5
	OBUDU	359	9
	ODUKPANI	221	5
	AKAMKPA	638	17
	BAKASSI	259	33
	BEKWARRA	262	10
	CALABAR SOUTH	444	9
	ETUNG	227	11
	OBUBRA	503	12
	YAKURR	594	12
	YALA	486	9
	CALABAR MUNICIPALITY	2472	54
	IKOM	1112	27
	OGOJA	822	19
**CROSS RIVER**	**Total**	**9838**	**13.6**
LAGOS	BADAGRY	716	12
	EPE	554	12
	IBEJU LEKKI	493	17
	IKEJA	2871	36
	OJO	1434	9
	AMUWO-ODOFIN	1218	15
	IFAKO-IJAIYE	2353	22
	IKORODU	1674	13
	KOSOFE	2273	13
	LAGOS MAINLAND	1642	20
	MUSHIN	2940	19
	OSHODI-ISOLO	3116	20
	SHOMOLU	1706	17
	AGEGE	3835	33
	AJEROMI IFELODUN	3333	19
	ALIMOSHO	5684	17
	APAPA	2694	48
	ETI-OSA	2925	41
	LAGOS ISLAND	1772	33
	SURULERE	3457	27
**LAGOS**	**Total**	**46691**	**20.5**
NASARAWA	AWE	517	18
	KEANA	625	31
	TOTO	368	12
	AKWANGA	947	34
	DOMA	1067	31
	KEFFI	1882	81
	KOKONA	1486	55
	LAFIA	3695	45
	NASSARAWA	1191	25
	NASSARAWA EGGON	852	23
	OBI	1393	37
	WAMBA	526	29
	KARU	5404	100
**NASARAWA**	**Total**	**19953**	**42.7**
ONDO	AKOKO SOUTH-EAST	730	35
	ILAJE	860	12
	ONDO-WEST	2006	28
	OWO	1066	19
	AKURE-SOUTH	2687	30
	ODIGBO	2328	40
**ONDO**	**Total**	**9677**	**26.2**

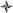
 Per 1000 adult men refers to men age >18 years of age.

### Estimates of FSWs by typology

The organizational typology of sex work differed substantially between the states. Table 5 shows that the most commonly identified typology across all included states was hotel or lodge-based FSWs, accounting for 32%, and this ranged from 23% of the FSWs in Cross River to 39% in Lagos. FSWs based in bars, night clubs, and casinos were the next largest category, accounting for 27% of the estimated number of FSW across the states and ranging from 13.7% in Nasarawa to 37.5% in Cross River. Overall, brothels accounted for 17% of FSW typologies, and ranged from 7.8% in Cross River to 26% in Ondo. Fifteen percent of FSWs were based in streets and other public places, and this ranged from 3% in Ondo to 29.8% in Nasarawa. Five percent of FSWs were classified as “other” and this included those who operate from home, trailer and truck stops, massage parlours, hostels and campuses, and escort services.

**Table 5:** Percentage distribution of FSWs by type of spot by state, Nigeria, 2012.

State	Type of spot						Total
	Brothel	Street/public	Home-based	Bar/night club/casino	Hotel/lodge	Other	
FCT	19.38%	17.63%	5.55%	26.77%	27.47%	3.19%	24,376
ANAMBRA	8.50%	17.72%	1.36%	33.97%	29.68%	8.77%	5,920
BENUE	9.97%	18.55%	6.48%	31.84%	28.33%	4.83%	10,034
CROSS RIVER	7.80%	22.27%	3.19%	37.58%	23.01%	6.16%	9,838
LAGOS	21.34%	5.94%	2.14%	26.91%	39.69%	3.98%	46,691
NASARAWA	10.09%	29.81%	9.25%	13.74%	29.55%	7.57%	19,953
ONDO	25.89%	3.02%	1.61%	38.23%	28.68%	2.58%	9,677
**Total (N%)**	16.98%	14.56%	4.27%	27.22%	32.23%	4.75%	126,489

### Findings from the venue profiling and rural appraisal

The other components of the appraisal revealed that 31.4% of the venues profiled had FSWs, and these venues accounted for 56% of the estimated number of males and females who frequent venues seeking casual sexual partners. While a large proportion of the venues in Lagos (55%), Benue (42%), Cross River and Nasarawa (38% each), Ondo (33%) facilitated FSW networks; this was the case for only 17% and 26% of venues in Anambra and FCT respectively. In the rural areas, more than one-third of the unmarried and married males reported ever having sex with a FSW with 14.5% and 13.6% of the unmarried and married males respectively reporting having sex with a FSW in the last 6 months.

## Discussion/Conclusions

To our knowledge this study was the first large scale attempt to geographically map the location of female sex worker populations in Nigeria and estimate their population sizes in the locations identified through the study. Our results show that there are large and diverse populations of FSWs with substantial geographic heterogeneity operating within the selected states. Understandably, Lagos, which is the commercial nerve center and most populous state in the country, has the largest FSW size estimate. However, when comparing the number of FSWs with the adult male population, the less populous states such as FCT, Nasarawa and Ondo, which have one-third the population of Lagos, have larger populations of FSWs relative to adult men. Similar results were also found in Pakistan and Kenya, both of which have undertaken mapping and size estimation of FSWs. In Kenya, the capital Nairobi also had the largest population estimates of FSWs. In Pakistan, Karachi, the most populous city, is home to the largest absolute number of FSWs. However, the less populous city of Nawabshah has the largest population of FSWs relative to men [7]. While the broad based classification of FSW typologies used in both countries is similar to the Nigerian context, the majority of the FSWs in Pakistan were found to be home-based while in Kenya and Nigeria more than half of the FSWs were found to be based in venues such as bars, hotels, and nightclubs) [7], [15].

The SACAs are finding the mapping data exceptionally valuable in the scale up of HIV prevention efforts within their states as it has enabled them to identify and describe locations where sex work is conducted at high densities, or ‘hot spots’ thus ensuring optimal distribution and reach of HIV prevention services at these sites. It has also enabled them to employ a more strategic and systemic approach in prioritizing locations for FSW interventions. This is clearly illustrated in FCT where Gwagwalada, a remote area council, an academic hub due to the presence of a university, but with few commercial activities is prioritized above AMAC, a more centrally located and commercially viable area based on the fact that it shows the most epidemic potential in relation to the population of FSW/per thousand men. This “LGA prioritization” process has been central to the development of the implementation roll out plan for the targeted FSW prevention programmes within the states. It ensures that locations where sex work may be a significant driver of the HIV epidemic in terms of the large FSW population or FSW density per thousand men are sufficiently saturated with interventions before other areas. Furthermore, in most of these states where majority of the HIV prevention response is donor driven, being the “gatekeepers” of this information has put the SACAs in the driver's seat on the coordination of implementation roll out among the multiple donor agencies implementing HIV prevention programmes for FSWs. This improved coordination effort has markedly reduced duplication of efforts among partners implementing programmes in the field.

Knowledge about the typologies and operational dynamics of female sex work within their states is also enabling the SACAs to determine which strategy to employ in designing targeted FSW programmes that are applicable to their own local context [7], [16]. States such as Nasarawa and Cross River, where a significant proportion of sex workers operate from street/public places emphasize peer outreach and provision of appropriate clinical services while states where most sex workers solicit at hotels/lodges and bars/night clubs (e.g. Lagos, Ondo, Benue) use alternate strategies such as engaging pimps, hotel staff and bar/night club staff to facilitate outreach and services. This is a novel approach in most of these states, as prevention programmes traditionally only targeted brothel based FSWs due to the impression that they constitute the majority of FSWs operating within states [6]. This has clearly been disproven as our data shows that out of the seven states mapped, only Cross River had a significant proportion of FSWs operating from brothels. In designing these targeted programmes, the SACAs have also taken the specific risk factors for the different sex worker typologies into consideration as the brothel based FSW have been found to have a higher HIV prevalence, higher number of clients and less correct knowledge about HIV transmission than the non brothel based FSW [6].

More importantly, the SACAs are using the mapping data to design an effective and cost efficient approach for program implementation as the information obtained is being used to determine the resources required to provide the necessary services to a high proportion of FSWs in a catchment area [17]. This information has enabled the SACAs to determine the cost of providing FSW interventions in particular hotspots thereby ensuring efficient allocation of resources to civil society organizations (CSOs) implementing FSW programmes in different parts of the state. This is exemplified in Anambra, where the cost of interventions for FSWs per spot in Akwa North with an FSW estimate of only 587 is significantly higher than in Akwa South with an FSW estimate of 1269 due to the fact that the average number of FSWs found per spot in Akwa North (15) is higher than Akwa South (9). It would have been impossible to make such detailed distinctions between both LGAs without the mapping data. The mapping goes even further by providing details about the specific typologies of sex work to be prioritized in each LGA. This information has enhanced the SACA's ability to effectively coordinate and monitor program implementation and thus prevent the “one size fits all” and “one off” approach normally used by CSOs implementing such HIV prevention programmes for FSWs [18]. Furthermore, the mapping data has been instrumental in the design of the proposed impact evaluation that will evaluate the impact of Nigeria's targeted HIV prevention program on averting new HIV infections among FSWs, their clients and communities. The mapping data served as the sampling frame for the random allocation of the units of intervention.

The SACAs are also using the information obtained from the Venue Profiling and Rural Appraisal to determine the types of “networking venues” to target. Given that there is significant overlap between FSW and casual networking sites across the states, the SACAs are targeting venues where men and women seeking casual partners frequent, as this would be an efficient strategy for targeting FSWs. In the rural areas, there has been an identified need to design specifically targeted programmes for unmarried and married men to address the high rate of high risk sexual behaviors exhibited from the appraisals.

Our geographic mapping approach has several limitations. First, because the geographic mapping methodology relies on numeric estimates rather than a count of FSWs at the spots identified this may lead to variability in the estimates derived. The methodology addresses this limitation by averaging estimates for spots identified by a large number of secondary key informants, and validating estimates for spots identified by the least number of secondary key informants through interviews with the FSWs themselves. Secondly, since the methodology is not individually based, it could overestimate the size of FSWs if they frequent multiple locations. However, since the methodology is rapid and focuses on the minimum, maximum and usual number of FSWs at a spot on a given day, the range of estimates (minimum to maximum) is unlikely to be skewed substantially. Moreover, the final estimates derived are adjusted to reflect the extent to which FSWs frequent multiple spots, based on primary key informant interviews. A third limitation is that the mapping methodology relies on secondary key informants to identify spots frequented by key populations. It is possible that some spots are missed and that females engaging in transactional sex or FSWs who are not venue based (such as those who contact clients using cell phones or social media) are likely not captured.

Despite these limitations, the geographic mapping approach has provided these states with an important baseline useful for scaling up all components of an integrated HIV prevention programme for FSWs. It will also enable implementers to utilize the cluster model of implementation – a highly effective implementation strategy whereby, a cluster of hotspots within each LGA is identified and linked to health facilities offering HIV testing, STI management, PMTCT and ART; and other key population-friendly services thereby ensuring saturation of the hotspots with the full minimum package of prevention interventions. It will enable programme managers of HIV prevention programmes for FSWs to determine, in a detailed way, which implementer is implementing which component of the FSW program in which hotspot. The use of such novel approaches that can accelerate the rapid scaling up of prevention programmes is imperative as programmes in several other settings have shown that a few interventions, implemented at sufficient scale, can markedly reduce the burden of HIV among sex workers and their clients and make considerable population-level impact [19], [20]. In the future, there is a need to further explore the socio-demographic and economic profiles and sexual behaviours of the FSWs by operational typology as well as understand their unmet needs in order to inform the design and implementation of even more comprehensive HIV prevention programmes for FSWs.
